# Color-coded molecular beacons for multiplex PCR screening assays

**DOI:** 10.1371/journal.pone.0213906

**Published:** 2019-03-18

**Authors:** Salvatore A. E. Marras, Sanjay Tyagi, Dan-Oscar Antson, Fred Russell Kramer

**Affiliations:** 1 Public Health Research Institute, Department of Microbiology, Biochemistry and Molecular Genetics, New Jersey Medical School, Rutgers University, Newark, New Jersey, United States of America; 2 Public Health Research Institute, Department of Medicine, New Jersey Medical School, Rutgers University, Newark, New Jersey, United States of America; 3 Center for Technology Licensing, Weill Cornell Medical Center, Cornell University, New York, New York, United States of America; University of Helsinki, FINLAND

## Abstract

The number of different fluorescent colors that can be distinguished in a PCR screening assay restricts the number of different targets that can be detected. If only six colors can be distinguished, and the probe for each target is labeled with a unique color, then only six different targets can be identified. Yet, it is often desirable to identify more targets. For instance, the rapid identification of which bacterial species (if any) is present in a patient’s normally sterile blood sample, out of a long list of species, would enable appropriate actions to be taken to prevent sepsis. We realized that the number of different targets that can be identified in a screening assay can be increased significantly by utilizing a unique combination of two colors for the identification of each target species. We prepared a demonstration assay in which 15 different molecular beacon probe pairs were present, each pair specific for the same identifying sequence in the 16S ribosomal RNA gene of a different bacterial species, and each pair labeled with a unique combination of two fluorophores out of the six differently colored fluorophores that our PCR instrument could distinguish. In a set of PCR assays, each containing all 30 color-coded molecular beacons, and each containing DNA from a different bacterial species, the only two colors that arose in each real-time assay identified the species-specific target sequence that was present. Due to the intrinsic low background of molecular beacon probes, these reactions were specific and extremely sensitive, and the threshold cycle reflected the abundance of the target sequence present in each sample.

## Introduction

When molecular beacon hybridization probes [1] are used to detect different target sequences in traditional PCR assays, each molecular beacon is labeled with a single uniquely colored fluorophore [2], and the number of different target sequences that can be identified is limited to the number of differently colored fluorophores that the PCR instrument can distinguish [3–5]. However, it is often desirable to be able to detect many more target sequences. For example, the identification of which species of bacteria is causing an infection, out of a relatively long list of bacterial species, enables an appropriate antibiotic to be used. We realized that this limitation can be overcome by utilizing combinations of labeled molecular beacons, in which the molecular beacon for each target sequence is prepared in two separate portions, and each portion is labeled with a differently colored fluorophore [6].

The advantage of such a duplex labeling scheme is that there are more unique combinations of exactly two colors than the number of colors that can be distinguished. For example, if the PCR instrument can distinguish six different fluorescent colors, then there are 15 unique combinations of two colors that can be utilized to label 15 different molecular beacon pairs, each pair specific for amplicons generated from a different target. As a result of this labeling scheme, one portion of the amplicons arising from a particular target sequence are labeled in one color and a second portion of the amplicons arising from the same target sequence are labeled in a different color. The combination of colors labeling the amplicons generated from a particular target sequence in the sample being tested serves as a code that unambiguously identifies the target that is present. This labeling paradigm is particularly useful for homogeneous, real-time PCR screening assays in which only one target sequence is likely to be present in the sample.

We prepared a demonstration assay, rather than an actual clinical assay, to illustrate the specificity and sensitivity of utilizing color-coded molecular beacons in multiplex, real-time PCR screening assays. This assay was designed to rapidly detect, identify, and quantitate 15 different bacterial species. The presence of any one of them in a normally sterile blood sample would indicate that sepsis could occur [7–9]. A pair of PCR primers that are specific for conserved sequences within the V3 region of the 16S ribosomal RNA gene of all 15 bacterial species was used to amplify the target sequences [10, 11]. In addition, 15 pairs of color-coded molecular beacons were present in the PCR assay mixture (30 different probes in all), each pair specific for a unique variable sequence that is present in the amplified DNA from one of the 15 bacterial species, and each pair labeled with a unique combination of two differently colored fluorophores out of the six differently colored fluorophores that the PCR instrument could distinguish. The results confirm that color-coded molecular beacon probes, by virtue of their hairpin structure can distinguish targets that differ by as little as a single-nucleotide polymorphism, and when they are not hybridized to their target, their fluorescence is quenched to such a great extent that many different color-coded molecular beacons can be present in a PCR assay without compromising the sensitivity of the assay.

## Materials

### Target plasmids

Cultures of 16 different sepsis-causing bacterial species were purchased from the American Type Culture Collection (Manassas, VA). The genomic DNA from each species was isolated and used as a template for the generation of copies of the V3 region of its 16S ribosomal RNA gene, utilizing the following PCR primers: 5’-ACTCCTACGGGAGGCAGCAGT-3’ and 5’-TATTACCGCGGCTGCTGGCAC-3’ (Integrated DNA Technologies, Coralville, IA). Each blunt-ended amplicon was cloned into the Srf I restriction site of a pPCR Script Amp SK(+) plasmid, utilizing a PCR-Script Amp Cloning Kit (Stratagene, La Jolla, CA). Target plasmids for use in the PCR screening assays were isolated from the transformed cells utilizing a CompactPrep Plasmid Kit (Qiagen, Hilden, Germany), and the plasmids were linearized by digestion with either Kpn I or Cla I restriction endonuclease, and their concentration was determined in a NanoDrop Spectrophotometer (ThermoFisher Scientific, Waltham, MA). The nucleotide sequence of the single bacterial gene segment inserted into each plasmid was confirmed by automated Sanger sequence analysis. These 16 linearized plasmids were used as targets for the demonstration assays (one of which served as a negative control to assess background fluorescence).

### Color-coded molecular beacons

Fifteen pairs of molecular beacon probes were synthesized, each pair specific for a unique target sequence present in a different bacterial species (Table 1). For designing the molecular beacons, the mfold Web Server was used to predict stem melting temperatures and to check that no internal stem-loop structures will form (http://unafold.rna.albany.edu/?q=mfold/DNA-Folding-Form); and the OligoAnalyzer 3.1 computer program was used to predict probe-target hybrid melting temperatures (http://www.idtdna.com/calc/analyzer). The molecular beacons were synthesized in our laboratory on a 394 DNA/RNA synthesizer (Applied Biosystems, Foster City, CA) utilizing β-cyanoethyl phosphoramidite precursors (Glen Research, Sterling, VA) on either Dabcyl controlled-pore glass columns (Glen Research) or on Black Hole Quencher-2 (BHQ-2) controlled-pore glass columns (LGC Biosearch Technologies, Petaluma, CA). The addition of fluorescein (FAM) and tetrachlorofluorescein (TET) was accomplished through the use of fluorophore-modified phosphoramidites; and the addition of other fluorophores was accomplished by incorporating an amino-modifier phosphoramidite at the 5’-terminal position of each oligonucleotide and then reacting those amino groups overnight in 100 mM NaHCO_3_ at 37°C with a succinimidyl ester derivative of one of the following fluorophores: Alexa Fluor 546, Alexa Fluor 568, Alexa Fluor 594 (all from ThermoFisher Scientific), or Atto 532 (from Sigma-Aldrich, St. Louis, MO). Each molecular beacon was purified on a System Gold High Performance Liquid Chromatograph (Beckman Coulter, Brea, CA) through a C-18 reverse-phase column (Waters Corporation, Milford, MA). A detailed protocol for molecular beacon synthesis is available at http://www.molecular-beacons.org.

**Table 1:** Uniquely color-coded molecular beacons.

Species	Sequence (5’ → 3’)	Relative Concentration
*Clostridium perfringens*	FAM–CGACGC–TCTTTGGGGAAGATAATGACGGT–GCGTCG–Dabcyl	1
	TET–CGACGC–TCTTTGGGGAAGATAATGACGGT–GCGTCG–Dabcyl	1
Streptococcus pneumoniae	FAM–CGACGC–TGGAAAGTTCACACTGTGACGGTAT–GCGTCG–Dabcyl	1
	Atto-532–CGACGC–TGGAAAGTTCACACTGTGACGGTAT–GCGTCG–Dabcyl	4
*Klebsiella pneumoniae*	FAM–CGCAGC–AGGAAGGCGGTGAGGTTAATA–GCTGCG–Dabcyl	2
	Alexa-546–CGCAG––AGGAAGGCGGTGAGGTTAATA––CTGCG–BHQ-2	4
Staphylococcus aureus	FAM–CGCAGC–AGTAACTGTGCACATCTTGACG–GCTGCG–Dabcyl	1
	Alexa-568–CGCAG––AGTAACTGTGCACATCTTGACG––CTGCG–BHQ-2	8
*Acinetobacter calcoaceticus*	FAM–CGACGC–GAGGAGGAGGCTACTGAAGTTAATA–GCGTCG–Dabcyl	1
	Alexa-594–CGCTG––––GGAGGAGGCTACTGAAGTTAATA––CAGCG–BHQ-2	12
Haemophilus influenzae	TET–CGACGC–AGGAAGGTTGATGTGTTAATAGTA–GCGTCG–Dabcyl	8
	Atto-532–CGACGC–AGGAAGGTTGATGTGTTAATAGTA–GCGTCG–Dabcyl	4
Clostridium difficile	TET–CGCACG–ACTCTGTCCTCAAGGAAGATAATG–CGTGCG–Dabcyl	1
	Alexa-546–CGCAC––ACTCTGTCCTCAAGGAAGATAATG––GTGCG–BHQ-2	4
*Klebsiella oxytoca*	TET–CGCTGC–AGGTTAATAACCTCAGCAATTG–GCAGCG–Dabcyl	2
	Alexa-568–CCGCG––AGGTTAATAACCTCAGCAATTG––CGCGG–BHQ-2	16
Neisseria gonorrhoeae	TET–CGACGC–GAAGAAAAGGCCGTTGCCAATATCG–GCGTCG–Dabcyl	1
	Alexa-594–CGCAG–––AAGAAAAGGCCGTTGCCAATATCG––CTGCG–BHQ-2	12
Enterobacter cloacae	Atto-532–CGCAGC–GGAGGAAGGTGTTGTGG–GCTGCG–Dabcyl	4
	Alexa-546–CGACG–––GAGGAAGGTGTTGTGG––CGTCG–BHQ-2	4
Campylobacter jejuni	Atto-532–CGCAGC–GCGTGGAGGATGACACTTTTCGGAG–GCTGCG–Dabcyl	2
	Alexa-568–CGCAG––GCGTGGAGGATGACACTTTTCGGAG––CTGCG–BHQ-2	8
*Serratia marcescens*	Atto-532–CGCAGC–CTTAATACGTTCATCAATTGACGTT–GCTGCG–Dabcyl	4
	Alexa-594–CGACG––CTTAATACGTTCATCAATTGACGTT––CGTCG–BHQ-2	12
Streptococcus agalactiae	Alexa-546–CGCAG–CGTTGGTAGGAGTGGAAAATCTA–CTGCG–BHQ-2	2
	Alexa-568–CGCAG–CGTTGGTAGGAGTGGAAAATCTA–CTGCG–BHQ-2	8
Neisseria meningitidis	Alexa-546–CGACG–AAGAAAAGGCTGTTGCTAATATCA–CGTCG–BHQ-2	2
	Alexa-594–CGACG–AAGAAAAGGCTGTTGCTAATATCA–CGTCG–BHQ-2	12
Staphylococcus epidermidis	Alexa-568–CGCAG–AGAACAAATGTGTAAGTAACTATG–CTGCG–BHQ-2	8
	Alexa-594–CGCAG–AGAACAAATGTGTAAGTAACTATG–CTGCG–BHQ-2	12

There are 15 unique combinations of six differently colored fluorophore labels. Emission maxima: FAM = 512 nm; TET = 536 nm; Atto 532 = 553 nm; Alexa Fluor 546 = 572 nm; Alexa Fluor 568 = 603 nm; Alexa Fluor 594 = 618 nm. The complementary arm sequences of the molecular beacons are underlined. In some probe pairs, the probe sequences are slightly different in length to assure similar melting temperatures. Also, to assure similar detection intensities, the concentration of each molecular beacon was adjusted, with the actual concentration being equal to the relative concentration x 13.3 nM.

### Polymerase chain reactions

Each 75-μL PCR assay contained 50 mM KCl, 6 mM MgCl_2_, 10 mM Tris-HCl (pH 8.0), 250 μM dATP, 250 μM dCTP, 250 μM dGTP, 250 μM dTTP, 3.75 Units of AmpliTaq Gold DNA polymerase (ThermoFisher Scientific), 50 nM limiting primer and 1,000 nM excess primer (Integrated DNA Technologies) whose nucleotide sequences are described below, 100,000 linearized plasmid DNA target molecules, and a 2,062 nM mixture of 30 different color-coded molecular beacons. The reactions were carried out in 200-μL white polypropylene tubes (USA Scientific, Ocala, FL) on an Applied Biosystems PRISM 7700 real-time spectrofluorometric thermal cycler, utilizing its Sequence Detector Version 1.6.3 analysis program to deconvolve the emission spectra. The reactions were incubated for 10 min at 95°C to activate the polymerase, followed by 40 cycles of 95°C denaturation for 15 sec, 58°C annealing for 30 sec, and 72°C chain elongation for 20 sec. The fluorescence intensity of each of the six fluorophores was measured during the 58°C annealing stage of each thermal cycle. The length of the PCR amplicons generated from each of the 16 different bacterial target plasmids varied between 162 and 188 base pairs.

### Design of the assays

#### Selection of the target sequences

The 16S ribosomal RNA gene of bacteria is a useful PCR target sequence for the identification of bacterial species because it includes sequence regions that are highly conserved from species to species, enabling a single pair of primers to be used for the generation of amplicons. These conserved DNA sequences encode portions of the ribosomal RNA to which ribosomal proteins bind. However, the sequences that lie between these conserved sites vary from species to species, providing an “identification bracelet” within the amplicons that can be read by color-coded molecular beacon hybridization probes. We therefore designed a PCR assay that could distinguish 15 different bacterial species by virtue of the unique sequence present in the V3 region of their 16S ribosomal RNA gene. Plasmids were prepared that contained the 16S ribosomal RNA gene target sequence for each of the 15 bacterial species. Table 2 shows the PCR priming sites and the unique molecular beacon target sequence that is present within each target plasmid.

**Table 2:** Target sequences for the PCR screening assays.

Species	3’–––––Limiting Primer Target–––––––––––––Molecular Beacon Target––––––––––––––(Excess Primer Sequence)–––––5‘
*Clostridium perfringens*	3’––––CCTCCGTCGTCACCCCTTA–––––––––––AGAAACCCCTTCTATTACTGCCA––––––––––(CACGGTCGTCGGCGCCATT)––––5’
Streptococcus pneumoniae	3’––––CCTCCGTCGTCATCCCTTA–––––––––––ACCTTTCAAGTGTGACACTGCCATA––––––––(CACGGTCGTCGGCGCCATT)––––5’
Klebsiella pneumoniae	3’––––CCTCCGTCGTCACCCCTTA–––––––––––TCCTTCCGCCACTCCAATTAT––––––––––––(CACGGTCGTCGGCGCCATT)––––5’
*Staphylococcus aureus*	3’––––CCTCCGTCGTCATCCCTTA–––––––––––TCATTGACACGTGTAGAACTGC–––––––––––(CACGGTCGTCGGCGCCATT)––––5’
*Acinetobacter calcoaceticus*	3’––––CCTCCGTCGTCACCCCTTA–––––––––––CTCCTCCTCCGATGACTTCAATTAT––––––––(CACGGTCGTCGGCGCCATT)––––5’
Haemophilus influenzae	3’––––CCTCCGTCGTCACCCCTTA–––––––––––TCCTTCCAACTACACAATTATCAT–––––––––(CACGGTCGTCGGCGCCATT)––––5’
Clostridium difficile	3’––––CCTCCGTCGTCACCCCTTA–––––––––––TGAGACAGGAGTTCCTTCTATTAC–––––––––(CACGGTCGTCGGCGCCATT)––––5’
Klebsiella oxytoca	3’––––CCTCCGTCGTCACCCCTTA–––––––––––TCCAATTATTGGAGTCGTTAAC–––––––––––(CACGGTCGTCGGCGCCATT)––––5’
Neisseria gonorrhoeae	3’––––CCTCCGTCGTCACCCCTTA–––––––––––CTTCTTTTCCGGCAACGGTTATAGC––––––––(CACGGTCGTCGGCGCCATT)––––5’
Enterobacter cloacae	3’––––GCTCCGTCGTCACCCCTTA–––––––––––CCTCCTTCCACAACACC––––––––––––––––(CACGGTCGTCGGCGCCATT)––––5’
*Campylobacter jejuni*	3’––––CCTCCGTCGTCATCCCTTA–––––––––––CGCACCTCCTACTGTGAAAAGCCTC––––––––(CACGGTCGTCGGCGCCATT)––––5’
Serratia marcescens	3’––––CCTCCGTCGTCACCCCTTA–––––––––––GAATTATGCAAGTAGTTAACTGCAA––––––––(CACGGTCGTCGGCGCCATT)––––5’
Streptococcus agalactiae	3’––––CCTCCGTCGTCATCCCTTA–––––––––––GCAACCATCCTCACCTTTTAGAT––––––––––(CACGGTCGTCGGCGCCATT)––––5’
Neisseria meningitidis	3’––––CCTCCGTCGTCACCCCTTA–––––––––––TTCTTTTCCGACAACGATTATAGT–––––––––(CACGGTCGTCGGCGCCATT)––––5’
Staphylococcus epidermidis	3’––––CCTCCGTCGTCATCCCTTA–––––––––––TCTTGTTTACACATTCATTGATAC–––––––––(CACGGTCGTCGGCGCCATT)––––5’
Streptococcus pyogenes	3’––––CCTCCGTCGTCATCCCTTA––––––––––––––––––––––––––––––––––––––––––––(CACGGTCGTCGGCGCCATT)––––5’

Underlined nucleotides in the limiting primer target sequence differ from species to species, but form a base pair with the corresponding inosine in the limiting primer. The *Streptococcus pyogenes* plasmid served as a control in a screening assay that contained all 30 molecular beacon probes, but did not contain any molecular beacon probes for *Streptococcus pyogenes*.

### Design of the PCR primers and their non-symmetric concentrations

Two PCR primers were prepared: a “limiting primer” that was present in the assays at 50 nM, and an “excess primer” that was present in the assays at 1,000 nM. The purpose of utilizing non-symmetric primer concentrations is that both primers take part in the initial exponential amplification of the target sequence, but after the limiting primer is used up, the excess primer continues the linear amplification of one of the two amplicon strands, thereby providing single-stranded targets for the hybridization of the molecular beacons, with virtually no competition from the complementary amplicon strand [12,13].

In order to enhance the specificity of priming, and to minimize the possibility of primer-dimer formation, each primer was designed to form a hairpin structure in which the nucleotide sequence of its 3’ arm, as well as all or part of the sequence in its loop, was complementary to its priming site in the target DNA sequence [14–16]. The sequence of the excess primer was 5’-GTGCCTTACCGCGGCTGCTGGCAC-3’, where the underlined nucleotides form the hairpin stem; and the entire loop sequence and the 3’-arm sequence were complementary to the conserved priming site in every bacterial target DNA. The sequence of the limiting primer was 5’-ATTCCCggggccIGAGGCAGCAGTIGGGAAT-3’, where the underlined nucleotides form the hairpin stem; but in this primer only the capitalized nucleotides in the loop sequence and the nucleotides in the 3’-arm sequence were complementary to the priming site in the bacterial target DNAs. Also, the limiting primer contained two inosine nucleotides (“I”), which can form a base pair with any of the four common nucleotides [17], as the complementary nucleotide in the corresponding position in the priming site of each bacterial species sometimes varied (see Table 2). The additional nucleotides in the loop of the limiting primer (represented by lower case letters) were present to assure that the limiting primer formed a strong hybrid with the amplicons, despite the lowering concentration of the limiting primer as it was used up during exponential synthesis.

### Design of the color-coded molecular beacon probes

Fifteen pairs of color-coded molecular beacons were designed for use in the PCR screening assays (Table 1). The nucleotide sequence in the loop of each pair of molecular beacons was perfectly complementary to the unique target sequence present in the amplicons synthesized from that pair’s intended bacterial target sequence (Table 2). Each pair of molecular beacons was labeled with two uniquely colored fluorophores out of the six differently colored fluorophores that could be distinguished by the PCR instrument. In designing the molecular beacon set, in order to ensure specificity, our goal was to utilize molecular beacons that would hybridize to their targets and generate a fluorescence signal only if their probe sequence was perfectly complementary to their intended target. If the probe sequence in the loop was not complementary to an available target sequence within an amplicon, the molecular beacon was designed to retain its hairpin shape, to not bind to that target, and to remain dark.

The high specificity of molecular beacon probes that distinguishes them from conventional linear hybridization probes is due to their hairpin shape [18]. When a molecular beacon is hybridized to its target sequence, it forms a relatively rigid probe-target double helix [19] that prevents the arms of the hairpin from interacting with each other, separating the fluorophore from the quencher, resulting in a bright fluorescence signal in the characteristic color of the fluorophore. However, if there is a mismatch between the sequence in the loop of the molecular beacon and its target sequence, then the molecular beacon is more stable retaining its hairpin shape, in which its fluorescence is quenched by contact between the fluorophore and the quencher. As a consequence, molecular beacons can be designed so that they only form a probe-target hybrid if every nucleotide in the loop sequence is complementary to the corresponding nucleotide in the target sequence [20].

In particular, to ensure the high specificity of each of the 30 different molecular beacons included in the demonstration assay, the length of the probe sequence in the loop of the hairpin, and the length of the complementary arm sequences in the hairpin stem, were designed to exhibit a very similar stability under the conditions present during the annealing stage of each thermal cycle (which is the stage during which molecular beacon fluorescence was measured). Moreover, since the stability of the hairpin stem of a molecular beacon depends on the strength of the transient bonds formed between the fluorophore and the quencher [21], as well as the strength of the nucleotide pairs in the stem helix, molecular beacons possessing a BHQ-2 quencher (which forms strong bonds with fluorophores) only required a stem that was five base pairs long, whereas the stems of the molecular beacons possessing a Dabcyl quencher needed to be six base pairs long.

### Adjusting the fluorescence intensities of the molecular beacons in the set

The fluorescence intensity of each of the six fluorophores at the PCR annealing temperature is dependent on the nature and intensity of the light used to excite their fluorescence. We carried out our PCR screening assays in an Applied Biosystems PRISM 7700 real-time spectrofluorometric thermal cycler, because this instrument can distinguish six different fluorescent colors. However, in this instrument fluorescence is excited by a blue argon ion laser, which efficiently stimulates green and yellow fluorescence in fluorophores such as FAM, TET, Atto 532, and Alexa Fluor 546, but is less efficient at stimulating orange and red fluorescence in fluorophores such as Alexa Fluor 568 and Alexa Fluor 594. Consequently, in preparing the mixture of molecular beacons to use in the demonstration PCR screening assays, we adjusted the concentration of each of the 30 different molecular beacons, utilizing more of those molecular beacons whose fluorophore is inefficiently stimulated by the blue laser and less of those molecular beacons whose fluorophore is more efficiently stimulated. The goal was to create a mixture in which the fluorescence intensity of the two molecular beacons in each pair was approximately the same. Table 1 lists the resulting concentration of each molecular beacon that was present in the PCR screening assays.

## Results

### PCR screening assays

**Fig 1:**
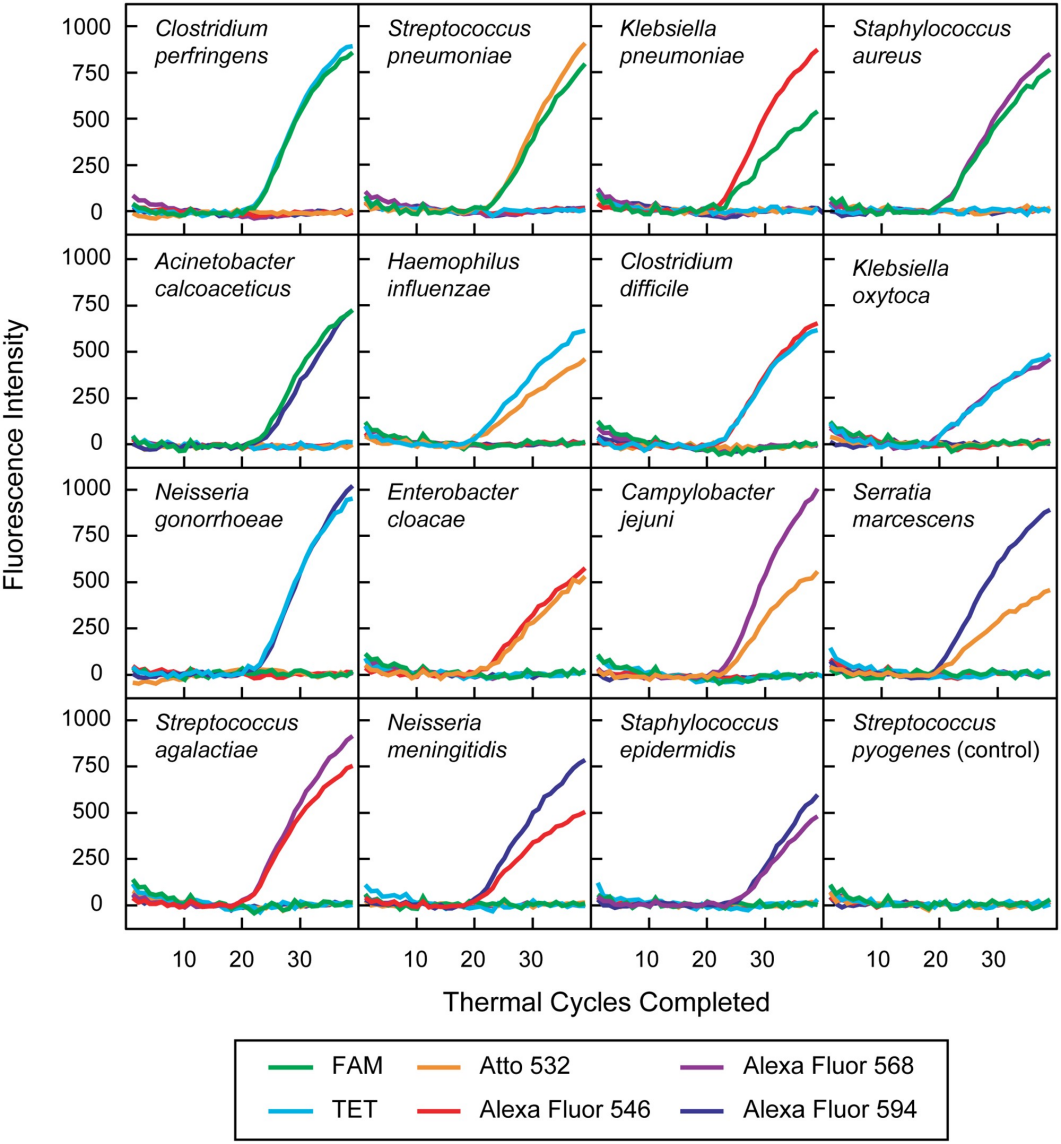
Screening assays. Real-time PCR assays containing 30 different color-coded molecular beacons were carried out, each assay initiated with 100,000 linearized target plasmids that contained a copy of the V3 region of the 16S ribosomal RNA gene from a different bacterial species. The unique duplex color combination that arose in each of the 15 screening assays unambiguously identified the species-specific target sequence that was initially present in each sample. A control assay containing a target plasmid from *Streptococcus pyogenes*, for which there were no molecular beacon probes, gave a very low background signal in all six colors, and remained low throughout the course of the assay.

Sixteen PCR screening assays were carried out, each initiated with 100,000 linearized target plasmids possessing the V3 region of the 16S ribosomal RNA gene from a different bacterial species. Each assay contained 50 nM of the limiting primer, 1,000 nM of the excess primer, and a mixture of 15 pairs of concentration-adjusted color-coded molecular beacons, each pair specific for the amplicons generated by a different bacterial species. One assay, containing target plasmids for *Streptococcus pyogenes*, served as an illustrative negative control, as there were no molecular beacons in the assay mixture that were specific for that bacterial species. Fluorescence was read during the annealing stage of each thermal cycle. The results are shown in Fig 1. Only two unique fluorescent colors (out of the six colors that the PCR instrument could distinguish) arose in each assay, and the particular combination of two colors uniquely identified each target species. No colors arose in the control assay, emphasizing the low background and excellent specificity of color-coded molecular beacon probes.

### Quantitation and sensitivity

**Fig 2:**
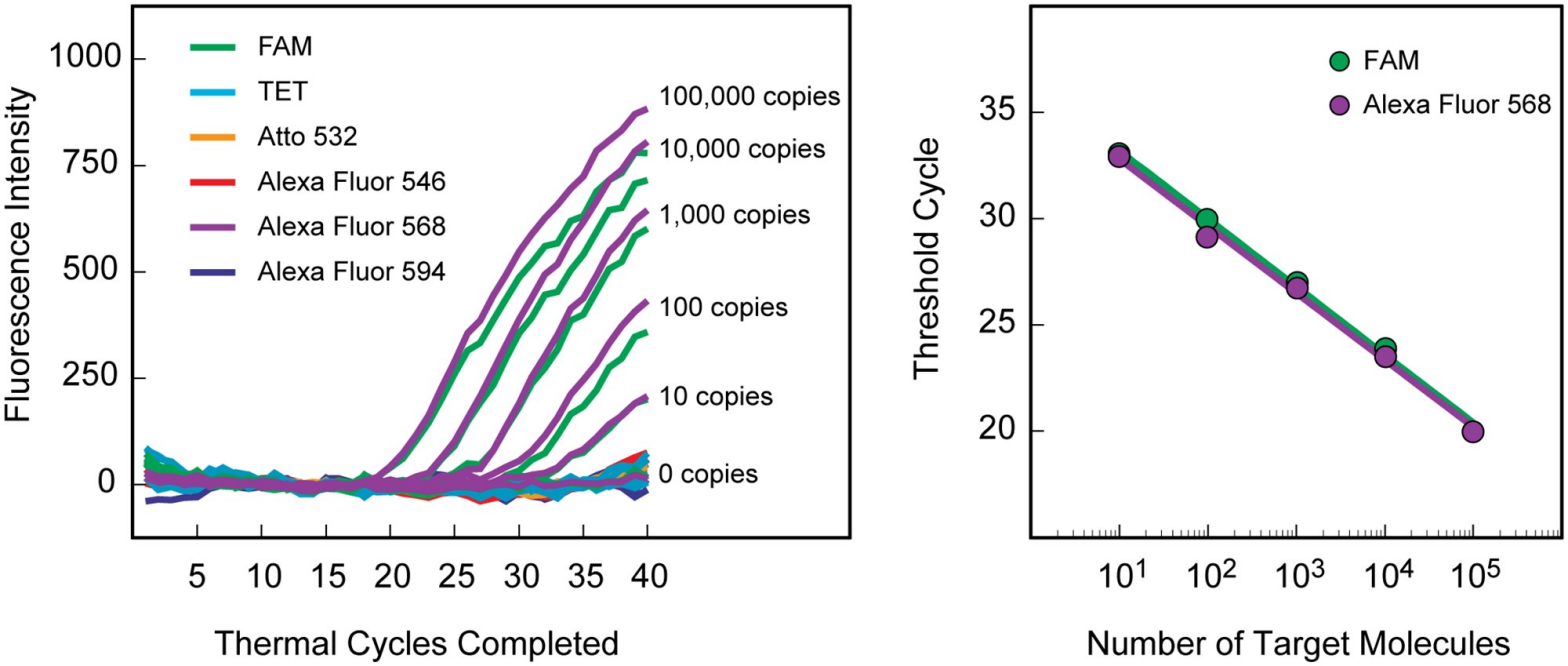
Sensitivity confirmation. Real-time PCR screening assays containing all 30 color-coded molecular beacons were initiated with different numbers of copies of the *Staphylococcus aureus* target plasmid. Fluorescent signals only arose from the two species-specific molecular beacons labeled with FAM and Alexa Fluor 568. The two-color threshold cycle of each reaction was inversely linearly proportional to the logarithm of the number of target molecules initially present in each sample. Low background signals from the 28 non-hybridized molecular beacons enabled the detection of as few as ten target molecules.

Six PCR assays were carried out, each initially containing a different quantity of *Staphylococcus aureus* targets (either 0; 10; 100; 1,000; 10,000; or 100,000 linearized plasmids), and each containing all 15 pairs of color-coded molecular beacons. The results are shown in Fig 2. Irrespective of whether there was initially a large number of targets or a small number of targets, only two of the six fluorescent colors arose, and they were the two colors that identified the targets as containing a sequence from *Staphylococcus aureus*. The fewer the number of targets initially present in the sample, the more thermal cycles were needed to be completed before the fluorescence signal arose above background (the threshold cycle). These results were in line with the classic relationship governing exponential amplification assays, in which the time it takes to generate a signal is inversely linearly proportional to the logarithm of the number of targets originally present in the sample [22, 23]. Most significantly, there was a clear signal from only ten target molecules, indicating that these real-time PCR screening assays, which utilize a mixture of 30 different color-coded molecular beacons, are as sensitive as traditional PCR assays that only possess a few probes.

## Discussion

### Intensity adjusted, uniquely color-coded molecular beacons

The PCR screening assay that we designed was a demonstration assay for the rapid identification of 15 different bacterial species that can cause sepsis, rather than an actual clinical assay, which would require extensive validation with clinical samples. Such a clinical assay would rapidly identify the species that is present in a blood sample (if any) in order to administer an effective antibiotic. Current practice is to send the blood sample out for culture (which takes a day or two) and to immediately administer a broad spectrum antibiotic (which may not be needed, and potentially could enable the growth of antibiotic-resistant strains throughout the body). Moreover, if the patient is infected, the broad spectrum antibiotic that is administered might be ineffective against the actual species that is present, and the patient could die of sepsis.

Our assay was designed to demonstrate the advantages of using uniquely color-coded molecular beacons in real-time PCR screening assays, enabling both the identity and the abundance of the target sequence to be determined. The resulting signal for each target is composed of a unique combination of exactly two colors. Moreover, the intensity of each member of the pair of identified colors is approximately equal, because the concentration of each differently labeled portion was adjusted by taking into consideration the intrinsic intensity of each fluorophore in the PCR buffer at the annealing temperature, and the efficiency of detection of each fluorophore by the particular PCR instrument in which the screening assay was carried out. Furthermore, the results demonstrate that the use of hairpin-shaped molecular beacon probes assures that background fluorescence is extremely low, enabling sensitive detection of the target sequence.

We purposely utilized the same number of colors (two) to label the molecular beacons for each target in our assay. The advantage of using only molecular beacons that are divided into the same number of portions is that the expected outcome is a signal composed of the same number of different colors (or the absence of a signal, should no target be present in the sample). Consequently, if more than the expected number of colors occur, that result would indicate that two or more different targets are present in the sample. However, when utilizing two colors for each target, if the sample unexpectedly contains two different targets whose identity is ambiguous, and the resulting signal consists of three or four colors, their identities can be unambiguously resolved by running a second assay with the same sample that utilizes a different set of two colors for each of the 15 pairs of molecular beacon probes. With this alternative labeling scheme, the result will be a different set of three or four colors, which compared to the original set of three or four colors enables the identification of the species involved [6].

The results suggest that higher multiplicities can be achieved. Using an instrument that can distinguish six different fluorescent colors, and if three different molecular beacon probes are utilized for each target, and if each of these three probes bears a differently colored fluorophore, then the occurrence of a signal containing that unique combination of three colors will indicate that the target of that probe set is present in the sample. Since there are 20 different unique combinations of three colors that can be assembled from the six colors that the instrument can distinguish, the screening assay will be able to identify 20 different target sequences. Although this triplex-coding arrangement would require 60 different molecular beacons, our results suggest that there should be no loss of sensitivity if they are well-designed. Moreover, if the PCR instrument can distinguish seven different fluorescent colors, then the number of targets that can be distinguished when three differently colored molecular beacons are used to identify each target is 35.

Earlier designs for enhancing the multiplicity of screening assays that employ combinatorially labeled hybridization probes that intermix singly labeled probes with probes labeled in different numbers of colored portions (and whose signal intensities were not adjusted) [24, 25], generate results that are more difficult to interpret, especially if multiple targets are present in the sample. Alternative schemes for enhancing the multiplicity of detection, that employ combinations of melting temperatures from differently colored probe-target hybrids to identify the target [26, 27], are carried out at end-point and are not able to determine the concentration of the target in the sample.

Detecting each target with multiple molecular beacons in these multiplex assays may increase the cost of assembling the assay for the first time, however, if the assay is used over and over, this increased expenditure will become a minor fraction of the overall cost. Furthermore, since each sample will be assayed just once, rather than multiple times, the overall cost will be less.

### Multiplex digital PCR applications

Perhaps the most exciting application for adjusted, uniquely color-coded molecular beacons that are designed for use in screening assays, involves their inclusion in assays that are carried out on digital PCR instruments. In digital PCR assays, the entire PCR mixture, including the sample and the hybridization probes, is divided into a very large number of individual wells or droplets (sometimes more than a million picoliter-sized droplets), with the result that only one target molecule (or no target molecule) is present in each droplet; and then simultaneous PCR is carried out in all of the droplets, and the amplicons that are generated in each droplet are then identified by virtue of the colored fluorescence signals that arise [28]. Therefore, each droplet is a PCR screening assay that identifies the one target molecule (if any) that is present in that droplet. Moreover, and this is a key point, although the PCR assay carried out in each droplet is a screening assay for the one target molecule that may be initially present in the droplet, the overall digital PCR assay is multiplex, able to identify and quantitate all of the different targets that are present in a sample (the abundance of each being determined by the number of droplets that are lit up in the same unique color combination).

There is great interest in utilizing digital PCR to simultaneously detect and quantitate a number of different targets in a sample [29]. However, the use of digital PCR for multiplex assays has been limited, because currently available digital PCR instruments can only distinguish two or three different fluorescent colors [30]. However, that will soon change. Digital PCR instruments are being developed that can distinguish as many as six different fluorescent colors [31]. Once this occurs, it should be very exciting to include uniquely color-coded molecular beacons in the digital PCR assay mixtures for the simultaneous identification and quantitation of 15 or 20 different targets in a sample. These digital PCR assays should be sensitive, quantitative, and highly reliable.
